# Chronic illness as transformative activity

**DOI:** 10.1007/s11019-025-10260-z

**Published:** 2025-03-17

**Authors:** Victoria Paul

**Affiliations:** Department of Design, Media and Educational Science, University of Southern Denmark, Campusvej 55, 5230 Odense M, Denmark

**Keywords:** Autonomy, Chronic illness, Phenomenology of illness, Transformative activity, Transformative experience

## Abstract

Laurie A. Paul (2014) developed the concept of transformative experience. In describing transformative experience as an experience that is both epistemically and personally transformative, she argues that transformative experience challenges the traditional model of rational decision making. Her concept of transformative experiences has been expanded to the field of illness. It has been argued that illness is a transformative experience because it fulfills Paul’s criteria for a transformative experience (Carel et al. 2016; Carel and Kidd 2020). Conceptualizing illness as a transformative experience would have far-reaching implications for the agency and for the rational decision-making process of ill persons. In considering these implications, this article questions the assumption that illness is a transformative experience and proposes that illness, especially when it is chronic, can be a transformative activity, in the sense that Agnes Callard (2020), introduced us to the concept of transformative activity. The article argues that conceptualizing (chronic) illness as a transformative activity strengthens the ill person’s agency and ability to learn to live with the illness.

## Introduction

What is it like to be ill? This is the fundamental question phenomenology of illness (PHI) is concerned with. Based on the phenomenology of Edmund Husserl, phenomenologists, such as Carel, Svenaeus and Toombs are interested in the structures of the experience of illness, and they argue that the subjective illness experience is crucial for an understanding of what it means to be ill. Emphasizing that humans not only have a body but are a body, phenomenologists argue that illness does not only affect the physicality of the ill person but her lived experience as such. Thus, severe illness, and chronic illness changes the person substantially, and her biography can be divided into life before the diagnosis and life after the diagnosis. In line with these standard accounts of PHI, it has been argued that illness is experienced as a transformative experience (TE), in the sense that L.A. Paul (2014) introduced us to the concept of TE (Carel et al. 2016; Carel and Kidd 2020).

In this paper, I challenge the assumption that illness experience is a TE. I propose that illness, especially when it is a chronic disease can be experienced as a transformative activity (TA). In arguing that illness experience can involve a TA, I will refer to A. Callard (2020) who distinguishes between transformative revelations^1^ and transformative activities. To accomplish this, I will begin the article by presenting Paul’s concept of TE and the phenomenologists’ application thereof to illness. Then, I will investigate Callard’s concept of TA and suggest that illness is not necessarily experienced as a TE but can entail the experiences of a TA. Concluding, the article will discuss the implications of this proposition for the patient’s agency, autonomy, and rational decision-making ability.

## Paul’s concept of transformative experience

The concept of TE goes back to Paul (2014) who introduced TE in the context of rational decision-making theory. Using a variety of examples, such as eating a durian fruit, becoming a parent, or becoming a vampire, Paul argues that such experiences are transformative. A TE is firstly a radical new experience that secondly transforms the person who is having such an experience both epistemically and personally. TE is epistemically transformative because the person gains the knowledge only through the experience. A person who is not a parent does not know what it feels like to be a parent, regardless of having heard many descriptions of what it is like to be a parent. She needs to make the experience herself to know how it feels to be a parent. With such transformation not only the knowledge of the person transforms but also her personality, that is her core values and beliefs change through the experience. Being a parent can change individuals and their perception of their world.

Paul argues that in being both epistemically and personally transformative, TE poses serious challenges to traditional models of rational decision-making. In emphasizing the importance of subjective value^2^ in a decision-making process, she raises the question of whether it is possible to make a rational decision regarding a life-changing event that will be both epistemically and personally transformative. According to Paul (2014) a rational decision-making process is as follows: the person who must decide must first assess the different options. This involves evaluating the different outcomes for one’s life and weighing the expected values of each outcome against each other, considering the advice of experts, family, friends, etc., as well as imaginatively projecting oneself in a possible future that involves the transformation.^3^ Evaluating the different outcomes for one’s life is crucial part for an authentic choice, which means to choose something that involves and can be justified by current values and preferences, allowing the person to identify with the consequences of the decision she is about to make.^4^ According to Paul, authenticity is the “extension of who I am now, in a way that is consistent with the values of my ‘true self’” (Paul 2020, 20). The concept of TE challenges this authenticity because the person who makes the decision cannot assess the subjective value of the options because she does not know what it feels like to undergo that experience and how she and her core values may change through the experience. Thus, a person who is facing a TE cannot base the decision solely on subjective values and yet basing the decision solely on reasons that are rational according to other people, such as experts, family and friends does not seem sufficient either. Thus, according to Paul the concept of TE challenges a person’s ability to make a rational decision for major life-changing events. Because of TEs far reaching consequences for a person’s ability to make a rational decision, Paul’s concept of TE is a widely discussed topic within decision theory. Within the last few years, it has been used in contexts outside of decision theory, such as within the context of illness experience. In the following part, I will analyze whether illness can account as a TE.

## Illness as transformative experience

It has been argued that illness is as a TE because it fulfills the criteria of TE (Carel et al. 2016; Carel and Kidd 2020). Firstly, illness is something radically new that happens to a person as a life-disrupting event. Secondly, illness is both epistemically and personally transformative. Contrary to instances of voluntary decisions, which are the primary subject discussed in rational decision theory, illness is an example of TEs that are either involuntary or nonvoluntary.^5^ Carel and Kidd (2020) expand the concept to include voluntary as well as nonvoluntary and in-voluntary TEs. In emphasizing the nonvoluntary and involuntary dimensions of TE, they focus less on the problems TE poses for rational decision-making, but rather they emphasize the experiential dimension of a transformative event, such as illness (Hofmann 2024). To fully grasp the suggestion that illness is a TE, it is necessary to elucidate the standard accounts of PHI that reinforce the assertion that illness is a TE.

Referring to Edmund Husserl, phenomenologists distinguish between the physical body (Körper) and the lived body (Leib). As embodied beings, illness is changing not only the physical functions of the ill person but her whole identity and lived experience. In distinguishing between healthy people and ill people, phenomenologists argue that health is experienced as some state of normality, in which the body recedes in the background of the lived experience. Hence, the experience of health is the experience of the body’s transparency (Leder 1990), whilst in illness the body is experienced in a state of resistance and limitation. An illness causes a person to experience a lack of control over her own body and future (Toombs 1987). This experience of lack of control over the body results in the feeling of alienation and unhomelikeness (Svenaeus 2011). Referring to the philosophy of Heidegger, Svenaeus (2011) argues that humans are always already embedded in the world and through their actions they create meaning. Illness disrupts these meaning structures because the ill person can no longer engage in certain activities that fulfilled and structured her life before the illness. Thus, the ill person does not feel at home after her diagnosis. In this regard, phenomenologists describe illness experience as the experience of a biographical disruption (Bury 1982) that divides the life of the person into a life before the disease and in a life with the disease.

As discussed above, disruption of meaning can lead to the experience of alienation, and the feeling of unhomelikeness. In distinguishing between these aspects, Carel (2016) emphasizes that illness is a disruptive event which does not need to lead to the feeling of alienation etc., but that can, in some cases, lead to neutral or even positive effects on the ill person’s life. People can adapt to their illness in a way that doesn’t diminish their overall well-being. To live well within the confines of illness, means that in most cases the ill person alters her values and desires, and updates her goals and ambitions in light of the limitations and opportunities brought about by illness (Carel et al. 2016). The list of potential personal changes due to illness is not exhausted by these examples but, unfortunately, it would be beyond the scope of this article to discuss the different forms that such adaptions may take. For this article, it is necessary to acknowledge that illness can be personally transformative in many ways, which makes it impossible to predict how a person might change through her illness (Carel et al. 2016). Acknowledging that the personal transformations brought about by TEs can be positively or negatively valanced, Carel and Kidd (2020) significantly refine and expand the concept of TE.

As much as illness is personally transformative, it is also epistemically transformative (Carel et al. 2016; Carel and Kidd 2020). Especially when it comes to illness being a TE, the distinction between personally transformative and epistemically transformative is crucial. Through an illness, a person gains knowledge that she did not have before, such as, for example, how the disease affects specific parts of her body or how to manage the illness. However, such an epistemic transformation does not necessarily lead to a personal one. Only that a person learns what it is like to have an illness and how it affects her body does not necessarily mean that the person will also subsequently alter her values and everyday routines. This gap between epistemic transformation and personal transformation can explicate such phenomena as non-adherence. This is, when patients know what they ought to do but do not change their behaviors or everyday routines to improve their health. In expanding Paul’s concept of TE by establishing “mixed TE” (Carel and Kidd 2020), the authors emphasize that epistemic transformation cannot be reduced to personal transformation or vice versa.

Even though phenomena such as non-adherence exist, in most cases illness is both epistemically and personally transformative; the experience of alterations in a person’s physicality are very likely to lead to a person renegotiating her options and values. This relation between epistemic and personal transformation is in accordance with the phenomenological assumption that humans are embodied, and therefore illness affects not only the physicality of a person but her lifeworld as such. Thus, epistemic knowledge entails not only the theoretical knowledge that explains a disease in purely medical terms, but also the experience of the changes in one’s body. In emphasizing the first-person perspective, phenomenologists try to understand the implications that illness has for the lived experience. Understanding what it means to be ill is qualitatively different from explaining the biological procedures and causalities that are taking place in the course of a disease. In emphasizing the first-person perspective, phenomenologists argue that an illness can only be understood by the person who undergoes the illness. “[…] to know, fully and first-hand, what it is like to have a serious illness, to experience bodily failure, vulnerability, and anxiety, about one’s body and one’s life, one needs to have the experience itself” (Carel et al. 2016). Using the example of a physician who gets diagnosed with a severe disease at some point, phenomenologists aim to show that being ill transforms the person’s knowledge of what it means to be ill (Carel et al. 2016). That is, even physicians who routinely engage with patients of some illness can still have a radically transformed understanding of what it means to be ill when they experience the illness firsthand themselves. Hence, illness experience is epistemically transformative because the knowledge of what it means to be ill is only fully available through the illness experience itself.^6^

Framing illness as a TE, in the sense of Paul’s concept of TE, is in harmony with standard accounts of PHI. Illness is a disruptive event that occurs mostly nonvoluntarily or involuntarily to the person who gets transformed, both personally and epistemically, by the illness. Through illness the person experiences what it is like to be ill, thereby acquiring knowledge she would not have had without the experience. In addition to this epistemic transformation, a person’s values, goals, and ambitions might change through the illness, as well as her perception of herself and her surrounding world. Hence, illness transforms the ill person in ways that are impossible to foresee. The uncertainty and unpredictability in epistemic and personal transformations create the possibility of ill persons to adjust to their illness in ways they would have not anticipated before the diagnosis. Thus, the notion of illness as a TE can alleviate a person’s concerns and therefore needs to be communicated between physician and patient (Carel et al. 2016).

## Callard’s concept of transformative activity

In the previous part of the article, I outlined Paul’s concept of TE and the challenges it poses to rational decision making. Further, the proposal that illness qualifies as a TE was analyzed in the context of PHI. According to standard accounts of PHI, illness would be a TE because illness is a radically new experience that happens to the person as a disruptive event in her life. Furthermore, illness is both epistemically and personally transformative. In the following, I will challenge the assumption that illness is a TE and I will propose that in most cases, especially when it comes to chronic illness, it is a TA.

Agnes Callard (2020) developed the concept of TA and distinguishes between transformative revelations and TAs (Callard 2020). While Paul’s examples of TEs—like the example of becoming a vampire— are transformative revelations,^7^ aspiration (Callard 2018; 2020) is a TA. While the person who becomes a vampire passively receives the poison and waits for it to transform her, the aspirant transforms gradually through the aspirational actions, that are learning activities, in that the aspirant learns to become somebody with new values. While TEs and TAs differ regarding patiency and agency, both experiences involve the “agential criterion” and the “learning criterion” (Callard 2020). The agential criterion means that the person chooses to engage in the transformation in some way. A person who is undergoing a TE, might have chosen actively to undergo the TE but is passively involved in the transformation itself. However, a person who experiences a TA is actively shaping the transformation through her actions. She is the agent of her transformation. If the person who is experiencing the TA were to stop what she is doing, the transformation itself would end. Hence, the transformation cannot be separated from the person’s actions. Precisely, because the person is transforming through her actions, a TA unfolds over time and is experienced as a process that transforms the person gradually. Unlike the TE, which is a major life-changing event, the TA unfolds in time and is the result of many decisions that cumulate to the transformation instead of being the result of one major decision.

Through the transformation, the person gains new knowledge. Both TEs and TAs involve the “learning criterion” (Callard 2020). A person who is undergoing a TE is gaining new knowledge about what it feels like to be transformed into the new being. This new knowledge is informed by the transformation itself and not by the actions of the person. The person who chooses to become a vampire offers her neck to the vampire’s bite. Indeed, this facilitates a TE, through which she learns what it is like to be a vampire. But her act of presenting her neck is not learning in and of itself (Callard 2020). Thus, the person is learning something new through the TE, that happens to her, and not through the actions she is performing. This demonstrates that TAs differ from TEs. The person who is engaged in a TA is learning through her actions, which directly affect her learning process. The person is transforming through her actions and her actions are motivated by her will to change into a person with values that she does not yet has. By trying to become what the person wants to become, she performs actions from which she learns. The aspirant is learning through her aspiration. Thus, the person who is actively engaged in a transformation is “learning by doing” (Callard 2020: 154). Her learning is directly informed by her actions. If she would end her actions, not only would the transformation end, but she would also not learn anymore.

However, engaging in a TA does not necessitate that the agent’s actions are directed to a well-defined goal. Rather, it means “doing what one does not yet know how to do, for reasons one does not yet grasp” (Callard 2020: 154). Herein, aspiration differs from ambition, because aspiration means to learn something new instead of becoming better at things one already knows how to do (Callard 2018). As source of motivation for the person who is acting towards her transformation, Callard (2016; 2018) introduces “proleptic reasons”. Proleptic reasons are reasons that are “provisional” and “rationalize large-scale transformation pursuits” (Callard 2016). They entail the agent’s awareness that she does not yet have the values she wants to have, or that she is not yet the person she wants to be in the future. For this reason, she must change her actions to become the person with the values she aspires to have. By choosing to act upon proleptic reasons, the person herself is in a transition because she cannot grasp what she wants to become. The value system is not yet hers and is therefore abstract. Her aspiration is the basis for her transformative activities.

In summation, firstly, TAs fulfill the agential criterion, meaning that someone experiencing a TA is actively engaging in her transformation without fully grasping what the transformation will feel like. Secondly, TAs fulfill the learning criterion, in that the transformation is a learning process for the person who is learning by doing. Thirdly, the TA is experienced as a transformative process that unfolds over time.

## Chronic illness as transformative activity

Having unwrapped the concept of TA, I will argue that it is helpful to understand chronic illness^8^ as something that can be a TA. Understanding chronic illness as something that can develop into a TA strengthens the agential skills of the chronically ill patient and has implications for the patient’s autonomy and decision making.

In chronic illness, the ill person is diagnosed with a disease that is persistent and, in most cases, incurable. Being diagnosed with a chronic disease means that one’s life has changed drastically because one must live with an illness. Some chronic diseases are less severe than others and, depending on the severity, the disease’s effect on the life of the ill person varies. However, learning to live with the knowledge that one is not fully healthy is part of being diagnosed with a chronic disease. Because the chronic illness is part of the lived experience of the chronically ill, it affects the person on medical, psychological and social levels. Hence, part of treating a chronic disease means to enable the person to adjust to the illness in such a way that it can be integrated in her life while also maintaining well-being. Phenomenologists who are themselves chronically ill have written that the chronic illness can have positive effects on life, and adjusting to an illness enables the person to live with an illness and to be well (Carel 2016). “Health-within-illness” (Lindsey 1996) formulates the simultaneity of illness and well- being.

Conversely, the “disability paradox” (Albrecht and Devlieger 1999) states that, against common assumption, people with disabilities rate their well-being better than those without disabilities. There is not a straightforward correlation between illness and poor quality of life, because it is “quite easy to imagine a sad, yet otherwise ‘healthy’ individual, and vice versa” (Sholl 2015: 406). However, it is very likely that chronic disease, especially when it is severe, does impact the overall well-being of the person, especially in the first months following the diagnosis. Cancer patients show a higher risk of depression, anxiety, and suicidal thoughts within the first months after a diagnosis which is, in most cases, expected to subside within the first year after diagnosis (Cook et al. 2018; Henson et al. 2019; Niedzwiedz et al. 2019). On the one hand, this progression leads to the strong assumption that disease shatters people’s well-being drastically. On the other hand, it also clearly demonstrates the possibility of adapting to the disease.

As elaborated in PHI, illness shatters people’s lives. That is, they are aware that in illness, the relation between self and body is not harmonious. Although this experience does not only apply to illness, but it is also an important feature of the subjective illness experience. Adapting to a chronic illness means to learn to live with this tension, and to integrate the illness and its limitation in one’s life in such a way that one can be well although being diseased. Such adaption to an illness is a learning process that unfolds over time. Chronically ill persons can learn which medication works for them and how to integrate illness in their life. Since the course of illness differs from person to person, and the personal and social circumstances of an ill person are highly individual there is no single way how to adapt to an illness. Rather, every person ought to figure out what (medication) works best and how to integrate the illness in one’s life.^9^

Thus, becoming a person that has integrated the illness successfully in life is a learning process that unfolds over time. How it will feel to be a person that has adapted to an illness and what it entails is not all clear to the person throughout the learning process. Rather the person is figuring out how to incorporate illness into her life through her actions and decisions over time. A chronic disease is not only happening to the person and transforming her both epistemically and personally, but rather the ill person is actively involved in becoming a person that has adapted to the disease. The adaption process requires the ill person to act in such way that she becomes the person she wants to be in the future, namely, a person who has integrated the illness in her life. It is hard to imagine an adaption process without the active involvement of the patient. Thus, the ill person might experience the disease in the first months after diagnosis as a disrupting event, but due to persistence she has the possibility to transform herself into somebody who is able to cope with the disease.^10^

As a person begins to integrate chronic disease into her life, she will inevitably make a series of decisions in terms of coping strategies, and more. In biomedical ethics, a patient’s decision-making is bound to the patient’s capacity for autonomy. In short, standard accounts of autonomy argue that being autonomous means to understand the relevant information from the health-care practitioners, reflect upon it, weigh the different options and their outcomes, and to come to a decision based on one’s deliberation, free from control by others (Beauchamp and Childress 2019). Hence, being autonomous entails being rational to some extent. Thus, it is unsurprising that it has been argued that if illness is a TE in Paul’s sense, it might impact people’s rational decision-making ability, and thereby their autonomy (Hofmann 2024; Villiger 2024).

Traditional accounts in which autonomy is fully understood in terms of cognitive capacities for reason has been criticized (Meyers 2005; Lewis and Holm 2022). Lewis (2021) distinguishes between a patient’s capacity for autonomy and their exercise of autonomy. Exercising autonomy does not necessarily involve rational deliberation. Furthermore, decisions, especially if they occur in the healthcare context, can be based on emotions, feelings and values that might not be fully rationalized by the individual that is making the decision. It is very well imaginable that a chronically ill person chooses a treatment option that might not be the best option from a medical perspective, but that enables her to fulfill certain values that she already had before the diagnosis. In such cases, the decision might not be rational, but based on values and emotions that are authentic to the person’s self. In this regard, the decision would be an autonomous one.

If a person chooses to act upon feelings such as the desire to establish a sort of equilibrium between her ill body and her values, she might not deliberately think about it, but only retroactively realize that her action made sense regarding her value system and what was important to her to achieve. Thus, acting upon one’s feelings and value system without reflecting upon it can be seen as an autonomous act that is primarily defined by the agent’s authenticity. The agent acts upon her values and regarding her values. Even if the person is confronted with a future that she does not yet fully grasp, as in the case of what it means to become a person that successfully integrates the illness in her life, she can act upon her current values or upon the values she would like to achieve. As Callard emphasizes in her concept of TA, proleptic reasons are sufficient motivation for actions. Meyers’ (2005) argument goes in the same direction when she states that in exercising skills, one constitutes one’s authentic self. Thus, in the case of chronic illness, the chronically ill can act upon her values and the values she might not fully have yet but wants to achieve in the future. This might entail the wish to re-establish life as it was before the disease or to adjust one’s life in accordance with the disease. What is important is that the person can act upon her values without rationalizing them and still is acting autonomously.

Thus, conceptualizing chronic illness experience as a TA has the advantage that it allows the ill person to engage actively in the transformative process, rather than being passively transformed by the illness. In being actively engaged in the transformative process, the ill person can act upon her values and upon the values she wants to achieve. Her decisions cannot be the result of rational deliberation in the strict sense because she cannot grasp their outcome yet due to the transformative nature both epistemically and personally. Still, she acts autonomously if we understand autonomy not only in terms of rational deliberation but as something that occurs in a broader context and is influenced by the person’s “pre-reflectively experienced […] values” (Lewis and Holm 2022: 625). Being actively involved in the process of becoming a person who has learned to integrate the chronic illness into her life not only enhances the patient’s autonomy, but also allows her to recover a degree of control over her life.

However, the proposed conceptualization of chronic illness as a TA does not entail the exclusion of chronic illness as a TE. Rather the proposition that chronic illness can be a TA should be understood as an expansion of the suggestion that illness experience is a TE (Carel et al. 2016; Carel and Kidd 2020). Thus, the relation between illness as a TA and illness as a TE is not exclusive. Rather, illness as a TE can develop into a TA if the ill person is actively learning to adjust to the illness and to integrate illness in her life. Many phenomenologists of illness draw on Heidegger’s concept of *Dasein*,^11^ which he famously introduced in “Being and Time” (2006). Similarly, I will make use of Heidegger’s concept of Dasein to briefly elaborate the interrelation between illness as TE and illness as TA. As opposed to *Vorhandensein* (Heidegger 2006), which is the simple existence of objects, humans exist while also being able to critically question and interpret their existence. By interpreting their being within the world, humans make sense of their Dasein and of their world. Such understanding of Dasein emphasizes the active dimension that enables humans to be the actors of their existence.

However, within some accounts of PHI, the disruptive effects that illness has for Dasein are emphasized, in that illness shatters everyday meaning patterns of a person. Having been diagnosed with an illness, it is up to the person how she chooses to engage with it. On the one hand, she may actively engage with the illness, thereby integrating it into her life. She would actively reinterpret her Dasein and would actively participate in her transformation. Thus, a person’s Dasein is always already open to active transformations although it is up to the person, to decide if she takes actions.

On the one hand, she may allow the illness to remain alien to her, thereby ignoring, refusing, or suppressing the illness. Conversely to the former case, here the ill person’s Dasein would be disrupted by her illness. Her transformation initiated by the illness, and by its effects on her body, would feel like something that simply happens to the person. Thus, the person would be transformed by the illness without actively shaping such transformation.

Accordingly, illness can be both a TE or/and a TA. Moreover, as the elaboration on the adaption process has shown, chronic illness can be a TE that might develop into a TA throughout the ill person’s illness journey. Hence, chronic illness as a TE and chronic illness as a TA are not exclusive.

## Concluding remarks

When it comes to illness experience, it has been argued that illness is experienced as a TE in the way that Paul introduces the concept. Illness is experienced as a TE because it is a disruptive event that happens to the ill person and is both epistemically and personally transformative. In this article, I proposed that illness, especially chronic illness, in many cases, is experienced is as a TA because the patient takes an active role in learning to adapt to the illness. In this process the patient is actively shaping her transition into such a person, and therefore the illness is not experienced as a passive disruption, but as a procedural change. Conceptualizing the experience of chronic disease as something that can be experienced as a TA has implications for medical ethics principles such as autonomy and rational decision-making. If chronic disease is communicated to the patient as something that can be integrated into one’s life, the patient is actively encouraged to engage in this integration process, leading to an enhancement in autonomy. Furthermore, the decisions that the patient is making are based on proleptic reasoning, meaning that the values that the ill person wants to reach in the future provide a source of motivation for the patient’s actions. Therefore, the way the patient wants to live in the face of illness, and her value system is the basis for her rational-decision making. Being in dialogue with the patient about what matters to her and how to rearrange priorities in a life with an illness is crucial for the way chronic disease is managed.

In arguing for chronic illness experience as an experience that does not necessarily be a TE but that rather can develop into a TA, the active involvement of the patient and her agency is emphasized. Also encouraged is a patient’s capacity to exercise autonomy in a process that is both epistemically and personally transformative. Furthermore, by conceptualizing illness as something that can involve a TA, the implications that the epistemic and personal transformations have for the patient’s autonomy and decision-making are substantially different from those resulting from the conceptualization of illness as a TE. Rather than assuming a diminishment in the ill person’s autonomy and decision-making, TA is strengthening both an ill person’s autonomy and decision-making capacities.

## Footnotes

1 Since most of Paul’s examples of TEs are transformative revelations, I will use TE throughout the article, and I will imply that TEs are transformative revelations.

2 Subjective values are “experientially grounded values attaching to lived experience” (Paul 2015: 762). Thus, subjective values are foremost phenomenal values that Paul (2015) calls “‘what it’s like’ values” (762).

3 Villiger (2024) provides a detailed and comprehensive description of Paul’s account of the rational decision-making process.

4 It is important that the self that must choose is in accordance with the future-self. If the “ex-ante” beliefs and values of the self are different from the ones of the “ex post” self, a decision for such an “ex post” self may result in a form of *self-alienation* for the self that is making a choice against her current values and beliefs. This dilemma can arise when the values and beliefs of the persons around the person are different from her own beliefs, and she feels as if she should replace her judgements with theirs. In such cases, making a rational choice means to choose against one’s own will and that seems to be problematic (Paul 2020).

5 Hofmann (2024) argues that in some case disease is voluntary chosen, for example when it is the result of a certain lifestyle or of preventive treatment. According to Hofmann (2024) voluntary chosen diseases are especially interesting for questioning how the concept of TE affects rational decision making and autonomy in the healthcare context.

6 Although it is problematic to suggest that illness is only fully accessible through the first-person perspective, especially when it comes to medical health care in which a common ground of understanding is crucial for the medical treatment, it would be beyond the scope of this article to get more involved with a critique on the supremacy of the first-person perspective in standard accounts of PHI. For further discussion, Gergel (2012) argued that such dominance of the first-person perspective can result in some form of solipsism while Sholl (2015) and Klausen (2021) argued that it is important to consider the biological as well as the subjective experience of illness, especially when it comes to mental disorders when the subjective perspective might not be reliable.

7 As stated in the beginning of the article, I will speak of transformative experience (TE) in the following, implying that the TEs are transformative revelations.

8 This part of the article is explicitly focusing on chronic illness because of its persistence that is allowing for a transformative process that is unfolding in time. Since phenomenologists of illness focus on severe and chronic illness without distinguishing explicitly between illness and chronic illness, the later was included throughout the article, while writing about illness.

9 In a similar direction, Leder (2024) introduces the concept of “chronic healing” (74), which refers to the flexibility of healing strategies over time. As a phenomenologist, he develops healing strategies that originate in the somewhat tense relation between having a body and being a body, such as embracing, escaping the body, reconnecting the body to the surrounding, remaking the body and re-timing the body (Leder 2024). By using the example of a chessboard on which each square indicates a different way of dealing with illness, Leder (2024) elaborates that just as the chess player, the ill person can cope with her illness by choosing, developing, and renegotiating different healing strategies appropriate to the context at different times.

10 Illness can be understood as a *Schicksalsereignis* (Tengelyi 1999) which is translated in English as a *destinal event*. Illness as a destinal event is something that happens to the ill person, that disrupts the person’s meaning patterns and which urges her to create new meaning. If illness can be considered as such a destinal event, it could then play two distinct roles simultaneously. Illness can be thought of as both a TE which happens to a person and also an event which potentially induces a TA, in that the ill person actively creates new meaning while dealing with the illness.

11 See Nielsen (2022) for a detailed discussion of Dasein within PHI.
